# Infantile Paralysis: Vast Strides in Recent Researches

**Published:** 1914-01-17

**Authors:** 


### INFANTILE PARALYSIS.

### Vast Strides in Recent Researches.

Five years ago, as the editor of the New York *Archives of Pediatrics* very truly remarks,* little more was known of the nature of poliomyelitis, better known by the name of its disastrous sequela —infantile paralysis—than its original scientific describer (von Heine) had told the world sixty years before. The medical profession had, it is true, outgrown the persistent superstition that a fall from the arms of a careless nurse was the usual cause of the disease; this idea, as Biblical students know, is at least as old as the time of Mephibosheth. It was also recognised that epidemics of infantile paralysis sometimes occur; and the belief prevailed that it was of the nature of an infective fever. But no proof of these theories could be offered, and the high authorities one and all professed a complete ignorance of the natural history of the disease and of its dissemination.

There is no doubt that the epidemics which were reported and studied in various parts of the world about the end of last century and the beginning of this one did much to stimulate interest and research in poliomyelitis. Medin was amongst the earliest to draw attention to epidemics in Stockholm. Scandinavia for some reason is especially cursed with infantile paralysis, though small epidemics are not unknown in Britain. Caverly, Wickman, Charcot, and Strumpell also deserve honourable mention in this connection. But the marvellous progress of the last four years, which has cleared up many of the problems of this disease and left the remainder on the verge of solution, has been almost exclusively the work of Americans, to whom the scientific world ascribes full and ungrudged credit for their brilliant researches.

In 1909 it was proved by Landsteiner, Levaditi, Flexner, and Lewis that poliomyelitis could be produced in monkeys by inoculating them with some of the secretions from cases of recent human poliomyelitis. Monkeys thus infected displayed symptoms very similar to those of children, and yielded identical pathological results *post mortem*; for the disease is very fatal in monkeys, indeed much more so than in children, in whom the death-rate is about 10 to 15 per cent.

Then it was shown that the virus of the disease, whatever it might be, would pass through a Berkefeld filter and remain active. Thus all the microorganisms which had been hitherto under suspicion were cleared of complicity. Next the discovery was made that from practically all parts of the body the virus could be obtained; this changed the conception of the disease from that of an affection of the nervous system to that of a generalised blood infection whose after-effects were chiefly manifested in the spinal cord. This view fitted in with conceptions which had been formed some years ago of the spinal paralysis as more an accident in the course of the disease than an essential part of it; and presently the authorities began to suggest that possibly paralysis should be considered as an unusual sequela instead of a universal one. Recovered monkeys were then shown to be immune to further infection, and their serum was also proved to protect non-immune individuals. Then the serum of adult human beings who had suffered from infantile paralysis in childhood was tested; and this, too, was shown to be protective. Upon this and other experimental research was built up gradually the hypothesis that possibly many or even most adults are immune through unrecognised attacks in infancy which have caused constitutional disturbance only and have not been followed by any paralysis. Poliomyelitis may, it is thought, be in reality a very mild disease in the vast majority of cases; and on this view there must be enormous numbers of undetected cases. Most persons, it is supposed, show only very mild symptoms, while a few exhibit the paralyses which have given the disease its name, and a smaller number still actually die.

The next thing to unmask was the method of spread of the disease, and it was considered possible that "carriers" might exist, as in the case of diphtheria, typhoid fever, and other infective diseases. This view was supported by the discovery that the nasal secretions of patients who had suffered from the disease two years before might still harbour the virus, and it has even been found in the secretions of unaffected individuals who have been in contact with cases. On the other hand, efforts to trace some intermediate insect host have failed: flies, mosquitoes, bugs, lice, and ticks have been suspect, but none has been proved guilty.

A few months ago the important announcement was made that Flexner and Noguchi have actually isolated the living virus. Using a culture medium of human ascitic fluid with sterile sheep's kidney, and incubating in this a piece of virulent nervous tissue, they isolated minute globular bodies which grow in chains, pairs, and small clumps. These organisms fulfil "Koch's postulates," and may therefore be accepted as the cause of the disease. They have been cultivated and sub-cultivated to the twentieth generation. They have been procured from the tissues of animals affected with poliomyelitis. They have, upon inoculation, reproduced the disease in other animals, from whose tissues in turn they have once more been recovered. Whether this organism is a bacterium or a protozoon is not yet definitely decided; some of its characteristics are held by the authorities to ally it with the former rather than the latter. On the solid foundations of scientific knowledge thus patiently laid, it is to be hoped, even confidently expected, that some efficient methods of cure and of prevention will some day be built up. When it is remembered what great progress the last few years have brought forth, it is impossible to despond for the future.

* October, 1913, p. 721.

